# Comprehensive analysis of PRPF19 immune infiltrates, DNA methylation, senescence-associated secretory phenotype and ceRNA network in bladder cancer

**DOI:** 10.3389/fimmu.2023.1289198

**Published:** 2023-11-06

**Authors:** YaXuan Wang, Jinfeng Wang, JiaXing He, Bo Ji, ZhongQi Pang, JianShe Wang, Yang Liu, MingHua Ren

**Affiliations:** Department of Urology, The First Affiliated Hospital of Harbin Medical University, Harbin, China

**Keywords:** prognosis, immune infiltration, senescence, DNA methylation, ceRNA

## Abstract

**Background:**

Pre-mRNA processing factor 19 (PRPF19) is an E3 ligase that plays a crucial role in repairing tumor-damaged cells and promoting cell survival. However, the predictive value and biological function of PRPF19 in bladder urothelial carcinoma (BLCA) require further investigation.

**Methods:**

In this study, we utilized transcriptomic data and bladder cancer tissue microarrays to identify the high expression of PRPF19 in BLCA, suggesting its potential as a prognostic biomarker. To gain a better understanding of the role of PRPF19 in the immune microenvironment of BLCA, we performed single cell analysis and employed the LASSO method. Additionally, we examined the methylation profiles of PRPF19 using the SMART website. Our investigation confirmed the correlation between PRPF19 and BLCA cell senescence and stemness. Furthermore, we constructed a PRPF19-miR-125a-5p-LINC02693-MIR4435-2HG ceRNA network using the ENCORI and miRWALK databases.

**Results:**

Our comprehensive analysis reveals that PRPF19 can serve as a prognostic marker for BLCA and is significantly associated with various immune-infiltrating cells in BLCA. Moreover, our findings suggest that PRPF19 influences cellular senescence through the regulation of stemness. Finally, we developed a ceRNA network that has the potential to predict the prognosis of BLCA patients.

**Conclusion:**

We confirmed the prognostic value and multiple biological functions of PRPF19 in BLCA. Furthermore, the specific ceRNA network can be used as a potential therapeutic target for BLCA.

## 1 Introduction

Bladder cancer, positioned among the top ten most prevalent cancers globally, encompasses muscle-invasive bladder cancer (MIBC) and non-muscle-invasive bladder cancer (NMIBC). Despite the use of standard treatments such as radical cystectomy or concurrent radiotherapy (1), the prognosis for patients diagnosed with bladder cancer remains unfavorable. Therefore, it is crucial to conduct a comprehensive investigation into the underlying mechanisms that lead to the development and progression of bladder cancer. Such an analysis is of utmost importance in facilitating screening and preventive measures.

PRPF19, also referred to as pre-mRNA processing factor 19, functions as an E3 ligase, which regulates cellular damage repair (2). Furthermore, it helps to avoid cell cycle arrest and prolong cell survival (3, 4). E3 ubiquitin ligases play a vital part in various cellular procedures. They are responsible for protein degradation, controlling protein-protein interactions and substrate activation or inactivation. Dysfunctional E3 frequently leads to cancer development (5, 6). This potential link to cancer immunotherapy shows promise for treating cancer by targeting E3 ubiquitin ligases. PRPF19, an E3 ubiquitin ligase, promotes colorectal cancer liver metastasis through activation of the Src-YAP1 pathway via K63-linked MYL9 ubiquitination (7). PRPF19 functions as an E3 ligase, serving as a fresh therapeutic target gene for pathological cardiac hypertrophy to inhibit cardiomyocyte hypertrophy (8). The heightened invasiveness of neuroblastoma cells may be linked to PRPF19’s regulation of the Hippo-YAP pathway (9). Additionally, PRPF19 governs MDM4 mRNA splicing in order to alter p53-dependent cellular senescence (10). Moreover, PRPF19 has been discovered to advance the development of tongue cancer and alter resistance to radiotherapy (11). Nevertheless, there exists a deficiency of research on the correlation between PRPF19 and bladder cancer, leading to the need for further study.

In this work, we examined bladder cancer data from TCGA and GEO to gather clinical data and RNA-seq gene expression information (12). We identified the expression pattern and functional network of PRPF19 in BLCA using multidimensional analysis. In addition, infiltrating immune cells linked to PRPF19 that may be relevant for prognosis were found using a machine learning technique. We investigated the relevance of PRPF19 to senescence-associated secretory phenotype (SASP)-related genes in bladder cancer. We also looked into how methylation of PRPF19 affects bladder cancer. Finally, we anticipated the PRPF19-connected ceRNA network using PRPF19 as the primary molecule. This offers a foundation for figuring out potential PRPF19-related molecular pathways.

## 2 Materials and methods

### 2.1 Samples, datasets and antibody

RNA-sequencing expression profiles (level 3) and associated clinical information for BLCA were acquired from TCGA (13), and GEO databases. The TCGA database consists of 406 bladder cancer samples and 19 normal bladder tissues, while the GSE13507 data set includes 165 bladder cancer samples and 9 normal bladder tissues. Genetic alterations data were obtained from the TCGA database. Bladder cancer tissue microarrays were purchased from Shanghai Outdo Biotech Company, and a total of 59 cases were included. Samples with lost visits or incomplete information were deleted (patients with unclear clinical staging and lost to follow-up), and finally 56 bladder cancer samples (including 32 paired normal bladder cancer tissues) were included in the study. All patients were operated from May 2007 to November 2011, and the follow-up was in March 2014, lasting 2.3-7 years. The study was ethically approved and informed consent was obtained from the patients. PRPF19 antibody (ab126776) was purchased from Abcam.

### 2.2 Prognostic analysis

A log-rank test was used to examine the variations in survival rates between these groups. The findings of each variable (P-value, HR, and 95% CI) were presented in the form of a forest plot using the “forestplot” program after a multivariate Cox regression analysis.

### 2.3 Immunohistochemical analysis of PRPF19 expression in bladder cancer tissue microarrays

After dewaxing the microarray, endogenous peroxidase blocker was added dropwise to the tissue and incubated at room temperature for 10 minutes for antigen repair, then after sealing was completed PRPF19 antibody (Dilution ratio is 1:60) was added and incubated overnight at 4°C, washed with PRPF19 antibody and then incubated with the secondary antibody for 30 minutes, and then finally the color was developed, re-stained, and sealed. Finally, the staining results were independently scored by 2 pathologists and the intensity of staining was categorized as low, medium, and high (assigned a score of 1,2,3 in that order) and the range of staining was categorized as 0-25%, 26%-50%, 51%-75%, and 76%-100% (assigned a score of 1,2,3, and 4 in that order).Finally, the two results were multiplied to obtain a total score, with ≤6 being low expression and >6 being high expression.

### 2.4 Gene enrichment analysis

The R software’s limma package was used to study the mRNA with differential expression. The threshold for differential expression of mRNAs was set at “adjusted P 0.05.” ClusterProfiler is a R utility that evaluates the GO function of candidate mRNAs and enriches the KEGG pathway, giving information on target genes’ oncogenic involvement. Another use for the ClusterProfiler tool is gene set enrichment analysis (GSEA) (14).

### 2.5 Immune infiltration analysis and machine learning

The present study aimed to investigate the potential association between PRPF19 expression and immune cells by analyzing the Tumor Immunization Single Cell Center (TISCH) database (15). This analysis aimed to shed light on the potential effects of PRPF19 at the individual cell level. Furthermore, the XCELL approach was used to select crucial immune cells from a group of 38 immune cells utilizing the Least Absolute Shrinkage and Selection Operator (LASSO) (16). The Kaplan-Meier survival analysis was used to analyze the disparity in survival rates between the aforementioned groups. Additionally, a time-dependent receiver operating characteristic (timeROC) study was performed to assess the predictive accuracy of the model.

### 2.6 DNA methylation analysis

The UALCAN database was used to get data on the variance in PRPF19 methylation levels between bladder cancer and normal bladder tissues (17). In addition, the SMARP database offered valuable insights into the variations in expression and prognostic implications of methylation probes targeting PRPF19 in the context of bladder cancer (18).

### 2.7 Stemness score of tumor cells

The determination of mRNAsi, as developed by Malta et al., may be achieved by the use of the OCLR technique. The creation of the gene expression profile is informed by the mRNA expression signature, which encompasses a total of 11,774 genes. The study used Spearman correlations to assess the similarity of RNA expression patterns. The dryness index is bounded inside the interval [0,1] due to the computation of subtracting the minimum value from the highest value and then dividing the result by the maximum value (19).

### 2.8 CeRNA network analysis

The MiRWALK platform (20) was used in our study to examine the microRNAs (miRNAs) that have the ability to target PRPF19.The probable interaction between screened miRNAs and long non-coding RNAs (lncRNAs) was predicted using ENCORI (21). In addition, the subcellular localization of ceRNAs was investigated with the Genecards program. The building of the ceRNA network was carried out based on the ceRNA hypothesis, which posits a negative association between mRNAs and lncRNAs and miRNAs. This was supported by RNA expression analysis and total survival analysis of the BLCA cohort.

### 2.9 Statistical analysis

All statistical analyses were conducted using version 4.0.3 of the R statistical software. The Wilcox test was utilized to compare the statistical disparity of two groups. The Kaplan-Meier curves for survival analysis underwent log-rank test. A p-value less than 0.05 was deemed to be statistically significant. *p < 0.05, **p < 0.01 and ***p < 0.001.

## 3 Result

### 3.1 PRPF expression and mutation analysis in BLCA

**Figure 1:**
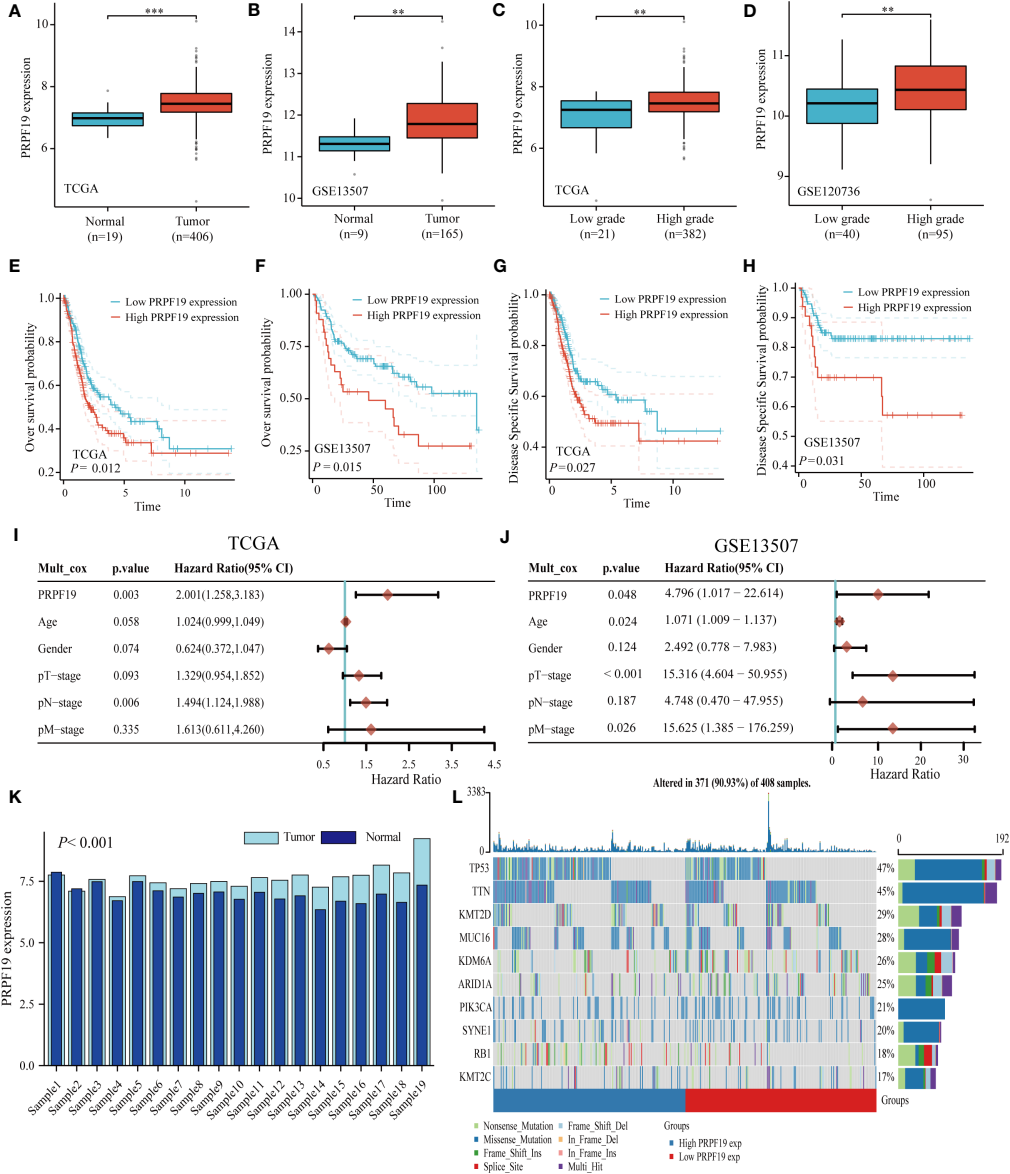
PRPF19 is significantly expressed in bladder cancer and is linked to high mutation frequency and a poor prognosis. **(A, B)** PRPF19 mRNA expression level in TCGA-BLCA databases and GSE13507 databases. **(C, D)** Expression levels of PRPF19 mRNA in low-grade and high-grade samples of TCGA-BLCA databases and GSE13507 databases. **(E, F)** Correlation between PRPF19 and overall survival in bladder cancer patients. **(G, H)** Correlation between PRPF19 and disease-free survival in bladder cancer patients. **(I, J)** Multifactorial COX regression analysis of the prognostic value of PRPF19 in bladder cancer samples. **(K)** Expression levels of PRPF19 in paired samples of bladder cancer in TCGA database. **(L)** Top 10 mutated genes in PRPF19 high expression and PRPF19 low expression groups from the TCGA database, shown as a heat map. **p < 0.01 and ***p < 0.001.

Initially, a comparative analysis of PRPF19 mRNA expression was conducted in bladder cancer and normal bladder tissues using the TCGA and GEO databases. The results showed a significant increase in PRPF19 mRNA expression in bladder cancer tissues compared to normal bladder tissues. This observation held true for both bladder cancer and GSE13507 primitive bladder tumor samples inside the TCGA database (**Figures 1A, B**). Notable alterations were seen in the expression of PRPF19 mRNA when comparing low-grade and high-grade bladder cancer samples (**Figures 1C, D**). Our analysis of the correlation between PRPF19 expression and the survival outcomes of bladder cancer patients, using the TCGA-BLCA dataset and the GSE13507 dataset, revealed that patients with high PRPF19 expression exhibited a significantly unfavorable prognosis in terms of both overall survival and disease-specific survival (**Figures 1E–H**). A multifactorial COX regression analysis was conducted, including age, gender, T-stage, N-stage, and M-stage as variables. The results of this analysis provided confirmation that PRPF19 serves as a valuable predictive biomarker for patients with bladder cancer (**Figures 1I, J**). PRPF19 mRNA levels were significantly higher in bladder cancer samples compared to paired paraneoplastic tissues in the TCGA bladder cancer dataset (**Figure 1K**). The present study used Oncoplot to visually represent the somatic landscape of the TCGA bladder cancer cohort, whereby samples were classified into high and low PRPF19 expression categories. The genes in question exhibited a higher frequency of mutations in the group with high PRPF19 expression, as shown by their ranking based on the number of mutations they underwent (**Figure 1L**). Therefore, it is plausible that PRPF19 might potentially have an impact on other genetic modifications and function as a prognostic indicator for persons afflicted with bladder cancer.

### 3.2 Validation of PRPF19 prognosis and expression in bladder cancer

**Figure 2:**
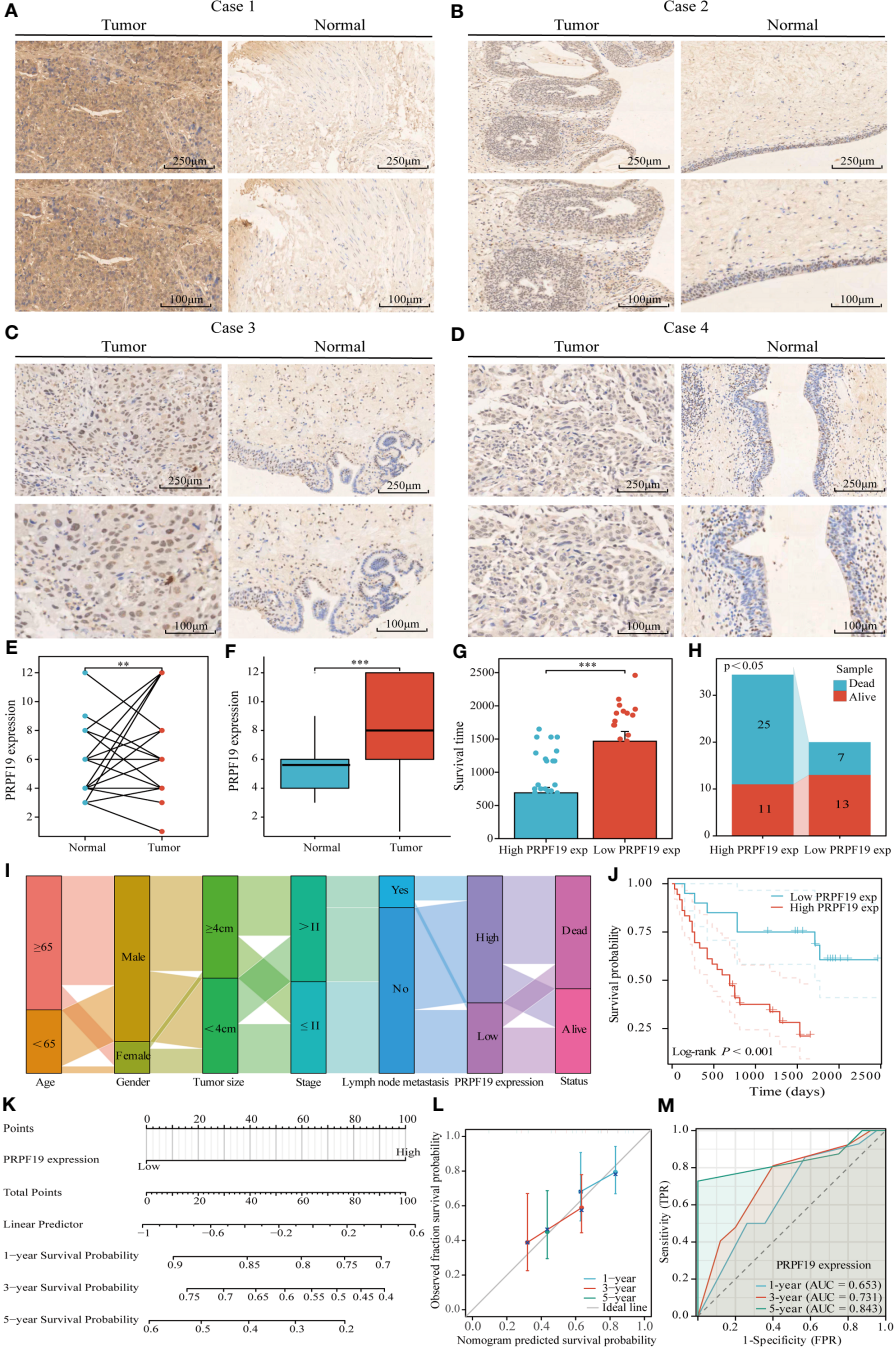
Tissue microarray analysis confirms high expression of PRPF19 in bladder cancer and its prognostic value. **(A–D)** Expression of PRPF19 in bladder cancer and normal tissues analyzed by immunohistochemical staining. **(E)** Expression of PRPF19 in paired bladder cancer tissues. **(F)** Expression of PRPF19 in 56 bladder cancer tissues and 32 normal bladder tissues. **(G)** Analysis of the difference in survival time between patients in the high PRPF19 expression group and low PRPF19 expression group. **(H)** Analysis of the ratio of survival/death of patients in the high PRPF19 expression group and low PRPF19 expression group. **(I)** Trends in the distribution of bladder cancer patients by age, sex, and other clinical factors. **(J)** Kaplan-Meier survival analysis of PRPF19 expression. **(K–M)** Analysis of the predictive ability of PRPF19 expression on the 1,3,5-year prognosis of bladder cancer patients. **p < 0.01 and ***p < 0.001.

To confirm the previously mentioned findings, we performed immunohistochemistry tests to investigate the expression and prognostic relevance of PRPF19 in bladder cancer. A set of 56 bladder cancer tissue samples and 32 normal bladder tissue samples were analyzed to further assess the expression and prognostic significance of PRPF19. The outcomes of our research are in line with bioinformatics-based investigations already conducted. Our observations reveal a noteworthy elevation in PRPF19 expression in bladder cancer tissues as against normal bladder tissues. (**Figures 2A–D**). In the context of paired bladder cancer tissue samples, it was shown that the expression of PRPF19 was markedly elevated compared to normal bladder tissue (**Figure 2E**). The difference in PRPF19 expression between bladder cancer and normal bladder cancer samples was shown by the use of box plots (**Figure 2F**). In this study, we conducted an analysis using **Table 1** to examine the relationship between PRPF19 expression and several clinical parameters in patients diagnosed with bladder cancer. The findings of our analysis provided confirmation that PRPF19 expression exhibited a significant association with both pTNM-stage and status. Following a comprehensive examination of the survival duration among patients categorized into the PRPF19 high-expression and PRPF19 low-expression groups, alongside an assessment of the ratio of survival to mortality, it was observed that patients in the PRPF19 low-expression group exhibited significantly prolonged survival periods compared to those with high PRPF19 expression (**Figure 2G**). Furthermore, it is worth noting that the mortality rate observed in the PRPF19 low expression group was much lower compared to the PRPF19 high expression group, as seen in **Figure 2H**. **Figure 2I** presents a Sankey diagram illustrating the distribution of high and low expression levels of PRPF19 in bladder cancer samples with respect to several factors such as age, gender, tumor size, stage, lymph node metastasis, and patient survival. **Table 2** presents the results of univariate COX regression analysis, which revealed significant associations between the prognosis of bladder cancer patients and variables such as pTNM-Stage, external aggression, and PRPF19. Furthermore, the findings from multivariate COX regression analysis indicated that PRPF19 holds potential as a prognostic biomarker for patients with bladder cancer. Patients exhibiting elevated levels of PRPF19 expression have a markedly worse prognosis, as seen in **Figure 2J**. In addition, it was ascertained that the expression of PRPF19 exhibited predictive capabilities for 1-, 3-, and 5-year survival in patients with bladder cancer, as shown by the utilization of nomograms and the assessment of area under the curve (AUC) (**Figures 2K–M**). In summary, our findings have substantiated the elevated expression of PRPF19 in bladder cancer and established its potential use as a predictive biomarker for individuals diagnosed with this malignancy.

**Table 1:** Correlation analysis of PRPF19 with pathological parameters of bladder cancer.

Characteristics	High	Low	P value
n	36	20	
Age, n (%)			0.147
≥65	22 (39.3%)	16 (28.6%)	
<65	14 (25%)	4 (7.1%)	
Gender, n (%)			0.588
male	29 (51.8%)	18 (32.1%)	
female	7 (12.5%)	2 (3.6%)	
Tumor size, n (%)			0.359
≥4	17 (30.4%)	12 (21.4%)	
<4	19 (33.9%)	8 (14.3%)	
Grade, n (%)			0.209
High	31 (55.4%)	20 (35.7%)	
Low	5 (8.9%)	0 (0%)	
pT-Stage T, n (%)			0.686
≥T2	27 (48.2%)	14 (25%)	
<T2	9 (16.1%)	6 (10.7%)	
pTNM-Stage, n (%)			0.038
>II	23 (41.1%)	7 (12.5%)	
≤II	13 (23.2%)	13 (23.2%)	
External aggression, n (%)			0.209
yes	5 (8.9%)	0 (0%)	
No	31 (55.4%)	20 (35.7%)	
Lymph node metastasis, n (%)			0.588
yes	7 (12.5%)	2 (3.6%)	
No	29 (51.8%)	18 (32.1%)	
Status, n (%)			0.013
Dead	25 (44.6%)	7 (12.5%)	
Alive	11 (19.6%)	13 (23.2%)	

**Table 2:** COX regression analysis of the prognostic correlation between PRPF19 and bladder cancer.

Characteristics	Total (N)	Univariate analysis		Multivariate analysis	
		Hazard ratio (95% CI)	P value	Hazard ratio (95% CI)	P value
Age	56				
≥65	38	Reference			
<65	18	1.338 (0.653 - 2.743)	0.427		
Gender	56				
male	47	Reference			
female	9	1.339 (0.547 - 3.276)	0.522		
Tumor size	56				
≥4	29	Reference			
<4	27	1.708 (0.838 - 3.483)	0.141		
Grade	56				
High	51	Reference			
Low	5	0.722 (0.171 - 3.041)	0.657		
pT-stage	56				
≥T2	41	Reference			
<T2	15	0.819 (0.367 - 1.830)	0.627		
pTNM-Stage	56				
>II	30	Reference		Reference	
≤II	26	0.418 (0.200 - 0.872)	0.020	0.640 (0.282 - 1.454)	0.286
External aggression	56				
yes	5	Reference		Reference	
No	51	0.355 (0.135 - 0.931)	0.035	0.768 (0.240 - 2.451)	0.655
Lymph node metastasis	56				
yes	9	Reference		Reference	
No	47	0.441 (0.188 - 1.033)	0.060	0.822 (0.290 - 2.330)	0.712
PRPF19 expression	56				
High	36	Reference		Reference	
Low	20	0.219 (0.082 - 0.580)	0.002	0.274 (0.099 - 0.758)	0.013

### 3.3 Enrichment analysis of PRPF19 in bladder cancer

**Figure 3:**
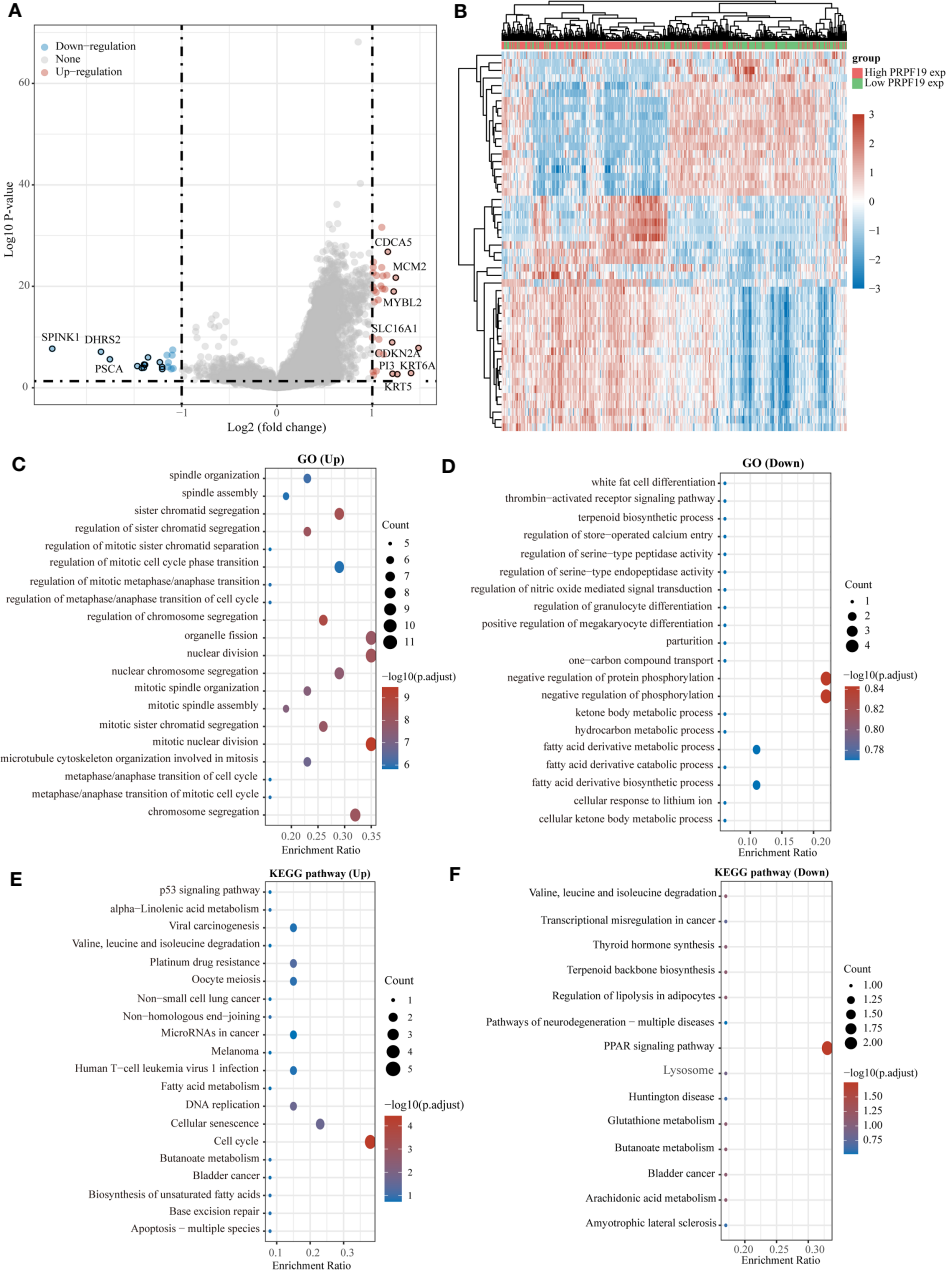
Analysis of the functions of PRPF19 in bladder cancer. **(A)** High-expression and low-expression subsets of PRPF19 were used in enrichment analysis and volcano mapping, respectively. **(B)** With the PRPF19 high expression and PRPF19 low expression groups, an enrichment analysis and a heat map were done. **(C, D)** Results of differential gene GO term enrichment. **(E, F)** Differential gene KEGG pathway enrichment results.

In the TCGA-BLCA dataset, samples from patients with bladder cancer were classified into high-expression and low-expression groups of PRPF19, based on the level of PRPF19 expression. The purpose of this study was to investigate the potential involvement of PRPF19 in bladder cancer (**Figures 3A, B**). The results of the GO enrichment analysis indicated that PRPF19 governs the regulation of vital biological processes, including nuclear division and the cell cycle (**Figures 3C, D**). The findings from the KEGG analysis demonstrate that PRPF19 has a regulatory role in several aspects of bladder cancer cell biology, including senescence, cell cycle progression, and resistance to platinum-based drugs (**Figures 3E, F**).

### 3.4 Gene set enrichment analysis of PRPF19 in BLCA

**Figure 4:**
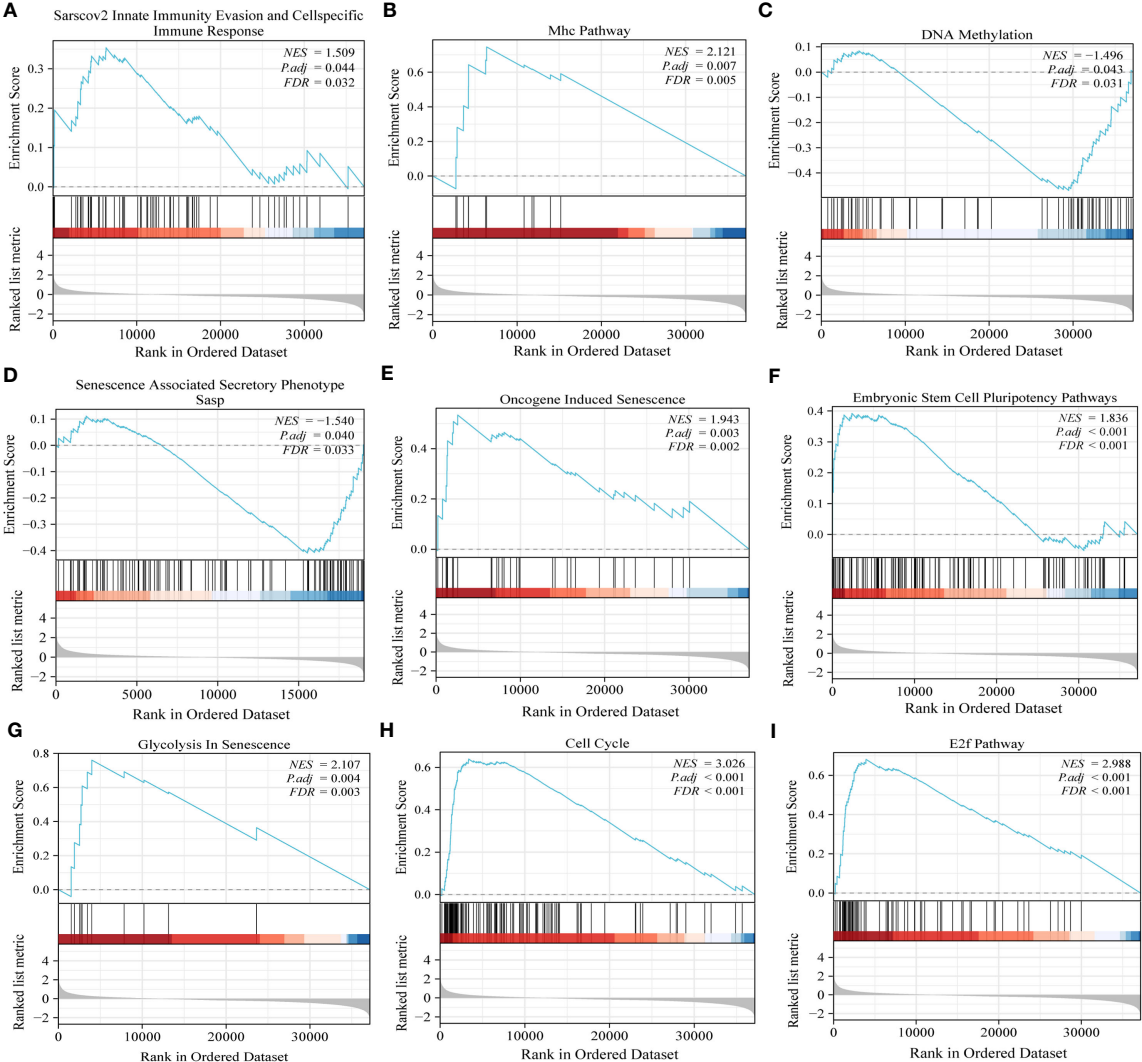
GSEA results confirm the involvement of PRPF19 in multiple biological functions of bladder cancer. **(A)** Sarscov2 innate immunity evasion and cellspecific immune response. **(B)** Mhc pathway. **(C)** DNA methylation. **(D)** Senescence associated secretory phenotype. **(E)** Oncogene induced senescence. **(F)** Embryonic stem cell pluripotency pathway. **(G)** Glycolysis in senescence. **(H)** cell cycle. **(I)** E2f pathway.

Gene set enrichment analysis (GSEA) offered more elucidation into the role of PRPF19 in the context of bladder cancer. The results obtained from GSEA demonstrated a statistically significant correlation between PRPF19 and Sarscov2 innate immunity evasion and cellspecific immune response, additionally, PRPF19 was shown to be associated with Mhc pathway (**Figures 4A, B**).T lymphocytes get activated and launch a targeted immune response upon the presentation of antigenic peptides by MHC molecules, resulting in the identification of both MHC molecules and antigenic peptides by T cells (22).Furthermore, recent research has shown evidence that the major histocompatibility complex (MHC) may function as a biomarker for immune checkpoint inhibitors, as indicated by many studies (23).Therefore, we posit that PRPF19 may have a substantial role in the immunological microenvironment of bladder cancer via the MHC route. In the interim, our examination of gene set enrichment further confirmed the noteworthy correlation between PRPF19 and various biological processes, including DNA methylation, senescence-associated secretory phenotype, senescence induced by oncogenes, pluripotency pathways in embryonic stem cells, senescence-related glycolysis, cell cycle, and the E2F pathway in BLCA (**Figures 4C–I**).

### 3.5 Correlation between PRPF19 expression and immune infiltration of bladder cancer

**Figure 5:**
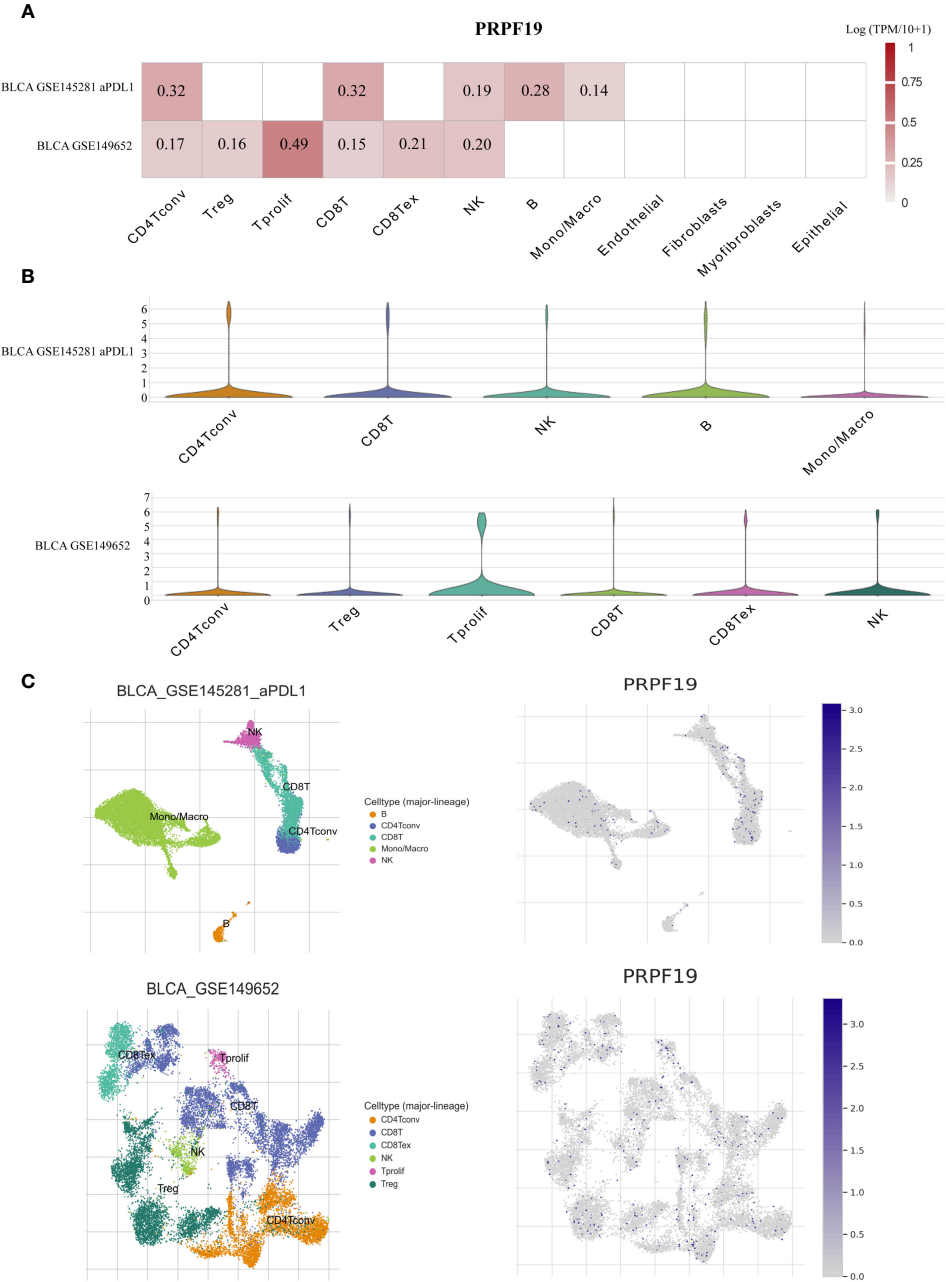
Investigating PRPF19 expression in BLCA’s various immune cells using scRNA-seq. **(A)** Independent scRNA-seq database heatmap showing PRPF19’s association with immune cell infiltration levels. **(B)** Violin plot of PRPF19 and immune cell infiltration. **(C)** Single-cell atlas including all of the cells that are included in the GSE145281 and GSE149652 datasets, as well as the expression and dispersion of PRPF19.

In order to examine the correlation between the distribution of immune cells and the expression levels of PRPF19 at the individual cell level, we obtained two distinct datasets related to BLCA from the scRNA-seq TISCH database (**Figure 5A**). Elevated levels of PRPF19 expression were seen in actively proliferating CD4 Tconv, CD8T, and NK cells within the BLCA GSE145281 aPDL1 and BLCA GSE149652 datasets, as shown in **Figures 5A, B**. The location and expression of PRPF19 in different immune cells were determined using clustered plots of single-cell RNA sequencing data (**Figure 5C**). The results of this study indicate a positive correlation between the expression levels of PRPF19 and the different kinds of immune cells seen in BLCA.

### 3.6 Analysis of key immune cells associated with PRPF19 in bladder cancer

**Figure 6:**
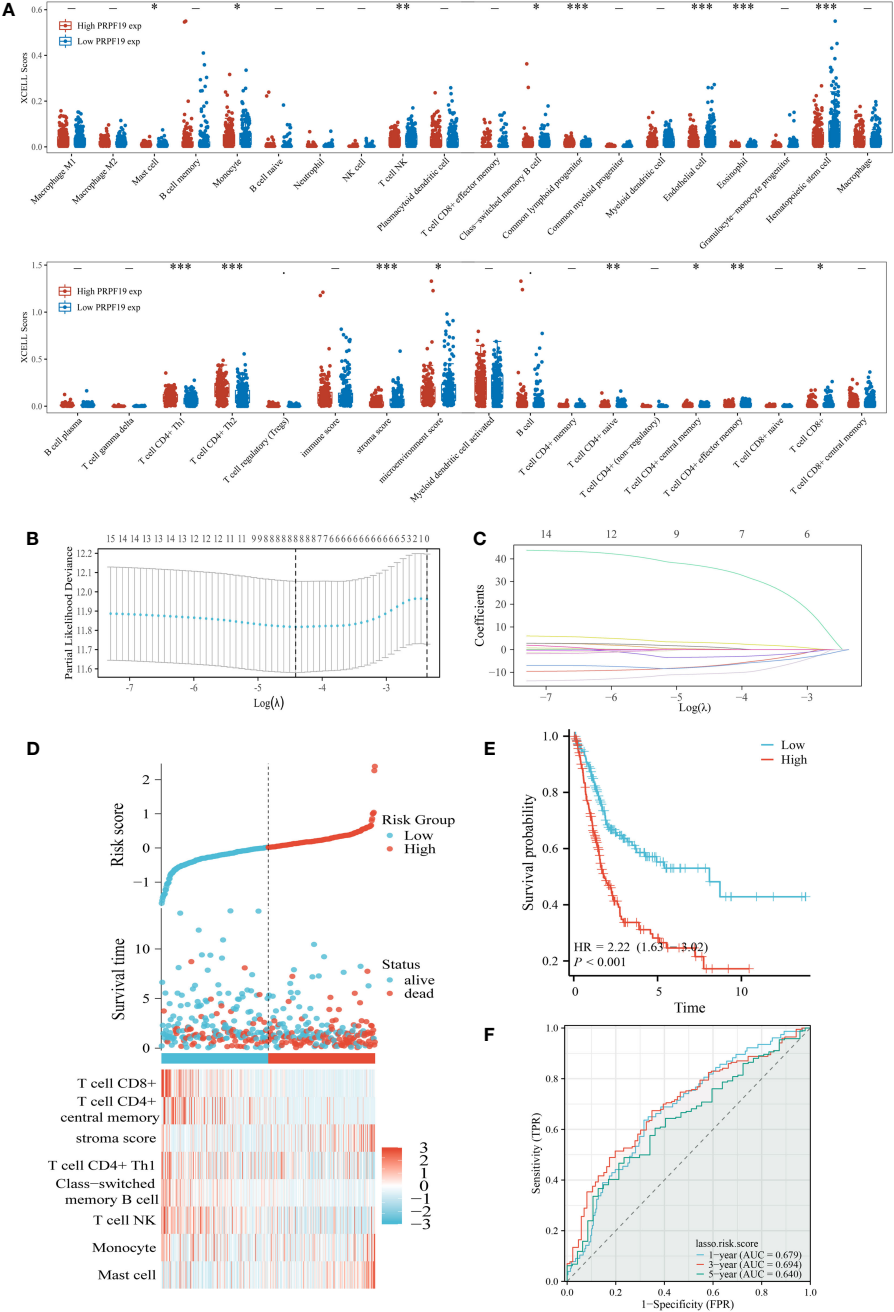
Identification of the key infiltrated immune cells by LASSO. **(A)** Immune infiltration varies across groups with high and low PRPF19 expression. **(B)** Diagram depicting penalties for 38 immune cells using the LASSO model. **(C)** Selective feature coefficients are shown as a function of the lambda parameter. **(D)** Eight prognostic immune infiltration-related genes and their expression patterns in BLCA patients with varying risk scores and survival outcomes. **(E, F)** ROC curves and overall survival curves for high- and low-risk BLCA patients, respectively. *p < 0.05, **p < 0.01 and ***p < 0.001.

The presence of immune cells inside tumors, known as immune infiltration, has a substantial influence on tumor development. The composition of immune cells residing in tumors has been acknowledged as a pivotal factor in determining the efficacy of cancer therapy (24). The XCELL deconvolution methodology was used to determine the relative abundance of 38 distinct immune cell types present within each BLCA sample. The examination of PRPF19 expression using subgroup analysis has shown a statistically significant correlation between PRPF19 and 16 discrete subcategories of infiltrating immune cells (**Figure 6A**). The LASSO technique was then used to develop prognostic models using a total of 8 of these 16 immune cells (**Figures 6B, C**). The risk score = (35.811)*Mast cell+(0.191)*Monocyte+(-7.033)*T cell NK+(-3.223)* Class-switched memory B cell+(1.052)* T cell CD4+Th1+(2.962)* stroma score+(-10.547)* T cell CD4+ central memory+(-7.270)* T cell CD8+. The BLCA patients were categorized into two groups based on their risk ratings. **Figure 6D** depicts the expression patterns of the eight genes over a range of risk scores, survival rates, and patient outcomes. Based on the Kaplan-Meier survival curves, it can be shown that patients belonging to the high-risk group had a less favorable outcome compared to those in the low-risk group (**Figure 6E**). The AUC values for the durations of 1, 3, and 5 years were seen to be 0.679, 0.694, and 0.640, respectively (**Figure 6F**).

### 3.7 Relationship between prognostic PRPF19 expression and critical immune infiltrate cells

**Figure 7:**
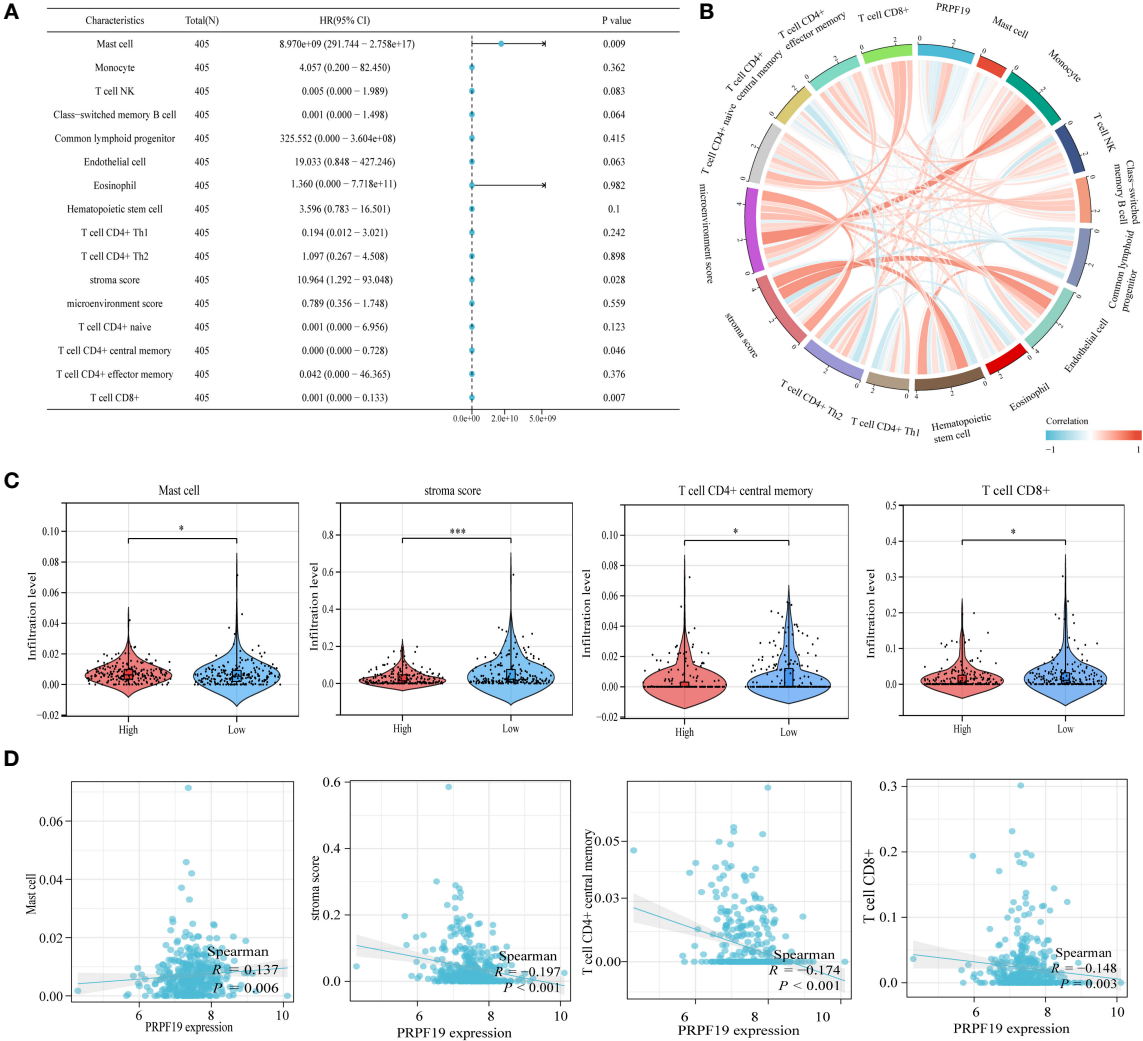
Validation and analysis of the persistence of the main infiltrating immune cells. **(A)** Analysis using univariate COX regression of the prognostic value of sixteen immune cells. **(B)** 16 immune cell correlation analysis. **(C)** Prognosis-related immune cell differences between the PRPF19 high and PRPF19 low expression groups. **(D)** Correlation of prognosis-related immune cell expression with PRPF19 expression. *p < 0.05 and ***p < 0.001.

The results of our univariate prognostic analysis, utilizing the major immune infiltrating cells collected from the previous section (**Figure 7A**), demonstrated a significant association between mast cell, stroma score, T cell CD4+ central memory, T cell CD8+, and prognosis in patients with BLCA. Subsequently, an examination was conducted to assess the connection among the 16 immune infiltrating cells. The results indicated that a significant proportion of these cells had a positive relationship with one another. This finding was substantiated by our examination of the association among the 16 immune invading cells, as seen in **Figure 7B**. Violin plots were used to demonstrate the notable dissimilarity in expression levels of the four prognosis-related immune infiltration cells between the PRPF19 high expression group and the PRPF19 low expression group (**Figure 7C**). In conclusion, we conducted a comparative analysis of the expression levels of four immune infiltrating cells and PRPF19. Our findings indicate a positive association between Mast cell and PRPF19 expression, whereas a negative correlation was seen between the expression levels of the other three immune infiltrating cells (**Figure 7D**).

### 3.8 An investigation of the relationship between the expression of PRPF19 and the methylation of DNA

**Figure 8:**
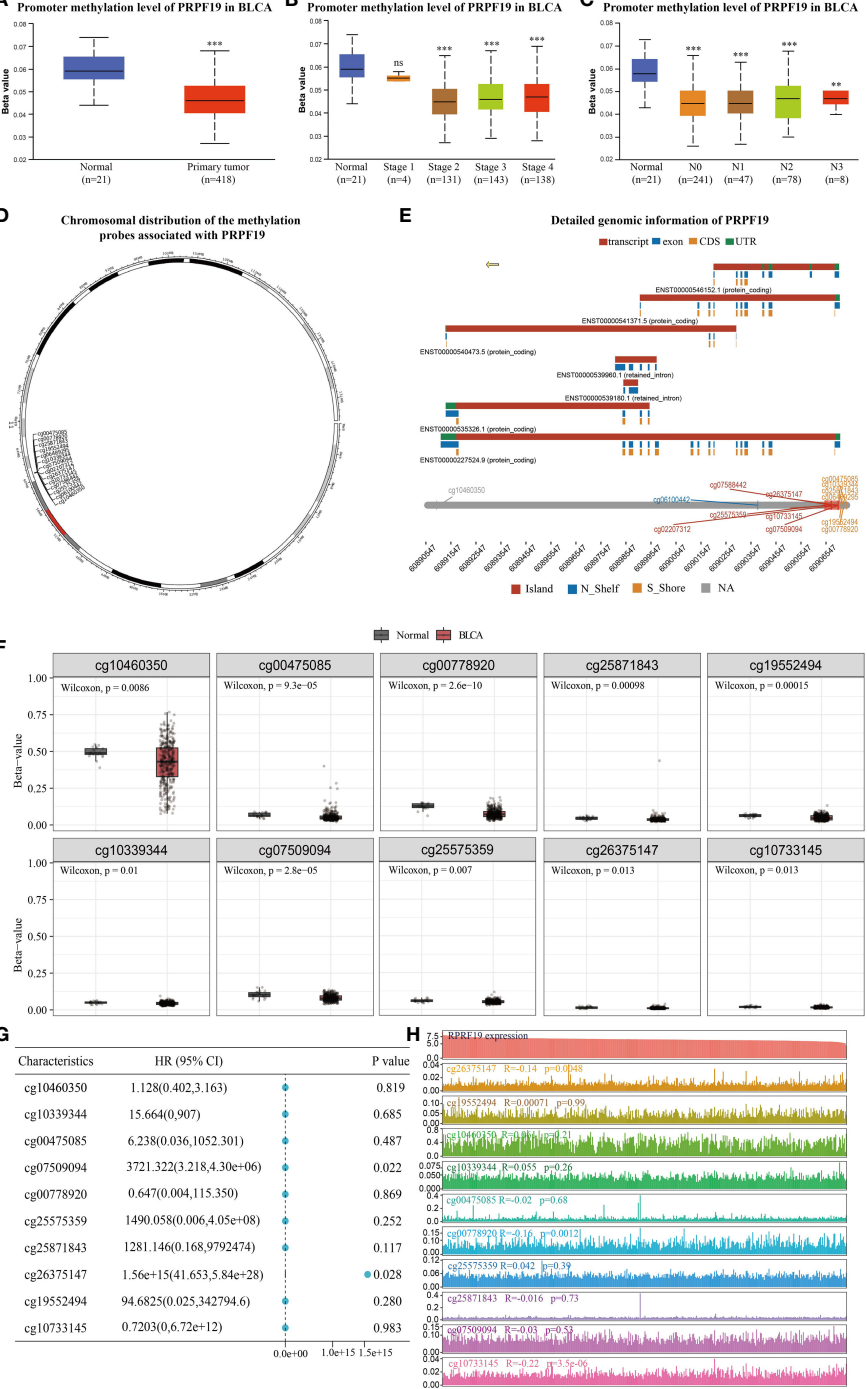
Analysis of PRPF19 methylation levels in bladder cancer. **(A)** Differences in PRPF19 methylation levels in bladder cancer and normal tissues. **(B)** Differential expression of PRPF19 methylation levels in different stages of bladder cancer. **(C)** Differential expression of PRPF19 methylation levels in different N stages of bladder cancer. **(D)** Methylation probes linked to PRPF19 and their chromosomal distribution. **(E)** Detailed genomic information of PRPF19. **(F)** Expression of different methylation probes in bladder cancer tissues and normal samples. **(G)** Prognostic value of different methylation probes in bladder cancer. **(H)** Expression distribution of different methylation probes in bladder cancer samples and their correlation with PRPF19 expression. **p < 0.01, ***p < 0.001 and ns not significant.

The UALCAN database was used to assess the presence of a statistically significant difference in PRPF19 methylation levels between bladder cancers and normal bladder tissues. It was observed that a difference existed between the methylation levels of PRPF19 in bladder tumors and normal bladder tissues. Specifically, the methylation levels of PRPF19 were shown to be much lower in bladder tumors compared to normal bladder tissues. This disparity became evident when analyzing bladder tumor patients at stage 2 of the disease (**Figures 8A, B**). Additionally, it was shown that the methylation levels of PRPF19 in bladder tumors of stages N0-3 were significantly lower compared to those in normal bladder tissue (**Figure 8C**). The SMART database was employed for analysis in order to investigate the involvement of PRPF19 methylation in bladder cancer. This analysis involved examining the chromosomal distribution of methylation probes associated with PRPF19, as well as exploring comprehensive genomic information related to PRPF19 (**Figures 8D, E**). The statistical analysis revealed a significant difference in the expression levels of 10 out of the 14 methylation probes between bladder cancer and normal bladder tissue, as seen in **Figure 8F**. The predictive relevance of the 10 differentially methylated probes in bladder cancer was then assessed, revealing a high association between cg07509094 and cg26375147 and the prognosis of individuals with bladder cancer (**Figure 8G**). Subsequently, the expression patterns of the aforementioned methylation probes in bladder cancer were successfully delineated (**Figure 8H**), followed by an investigation into their correlation with PRPF19 expression. The present study revealed a significant association between PRPF19 expression and the DNA methylation levels of cg26375147, cg00778920, and cg10733145.

### 3.9 Correlation analysis of PRPF19 with senescence associated secretory phenotypes

**Figure 9:**
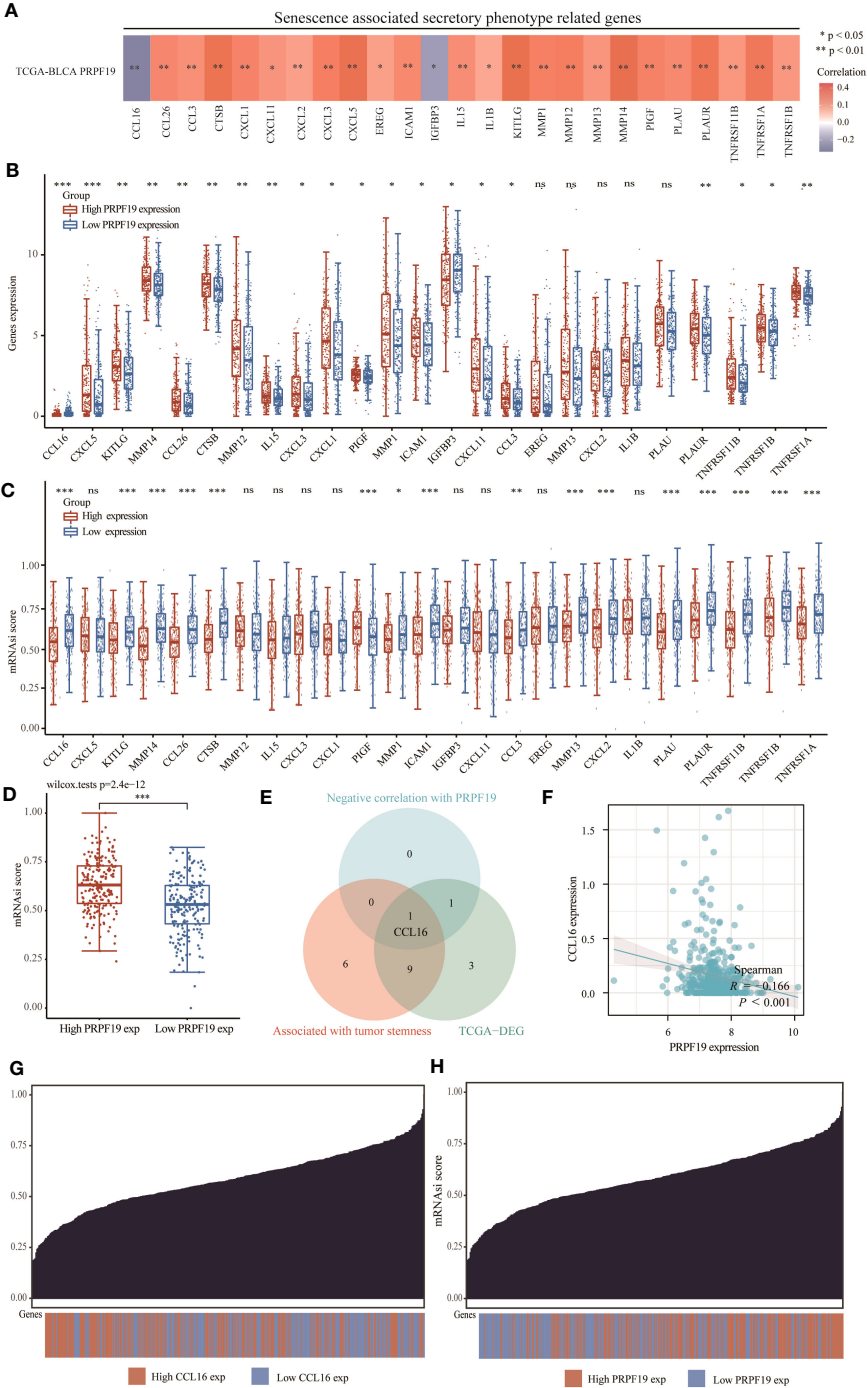
PRPF19 is significantly associated with senescence-associated secretory phenotypes. **(A)** Correlation analysis of PRPF19 with genes related to senescence-associated secretory phenotypes. **(B)** PRPF19 expression levels correlate differently with senescence-related secretory phenotypes. **(C)** Differential senescence-associated secretory phenotype genes positively associated with PRPF19 in the TCGA BLCA cohort and GSE13507 cohort. **(D)** Kaplan-Meier overall survival curves of CCL16. **(E)** Scatter plot of PRPF19 and CCL16 correlation. **(F)** Differences in stemness scores in bladder cancer samples between high and low PRPF19 expressing groups. **(G, H)** Distribution of stemness scores from low to high in bladder cancer samples.

Cellular senescence has historically been seen as a protective process that safeguards tissues against the development of cancer by permanently halting the cell cycle, restricting proliferation, and minimizing the transfer of unfavorable genetic alterations (25). Senescent cells refer to cancer cells that have ceased proliferating but retain their metabolic activity. These cells may lead to the adverse consequence of a modified cytosolic secretome, which is linked to localized inflammation, alterations in the extracellular environment, and heightened growth factor activity (26). In the first phase of our study, we conducted an investigation on the TCGA BLCA dataset to examine the potential correlation between PRPF19 expression levels and the expression levels of genes associated with SASP. The findings presented in **Figure 9A** revealed a negative correlation between PRPF19 and CCL16 as well as IGFBP3. The present study aimed to investigate potential disparities in the expression of genes associated with SASP across two distinct groups categorized based on their levels of PRPF19 expression. In addition to EREG, MMP13, CXCL2, IL1B, and PLAU, the results indicated significant differences in the expression levels of the remaining genes between the PRPF19 high expression group and the PRPF19 low expression group, as seen in **Figure 9B**. The genes associated with SASP were shown to be positively connected with a tumor stemness score. This observation suggests that senescent tumor cells could undergo reprogramming into tumor stem cells through this particular method (27). A robust association was seen between tumor stemness scores and a significant proportion of genes implicated in the secretory phenotype often linked to senescence (**Figure 9C**). Additionally, an investigation was conducted to examine the impact of PRPF19 on bladder cancer stem cells. The results revealed that cells with elevated levels of PRPF19 expression exhibited a greater stemness score compared to cells with lower levels of PRPF19 expression, as seen in **Figure 9D**. A single differential gene was identified to have a high correlation with the tumor stemness score and a negative association with PRPF19, as seen in **Figure 9E** via the use of Venn diagrams. The TCGA bladder cancer dataset revealed a significant correlation between the expression levels of CCL16 and PRPF19 (r=0.166) (**Figure 9F**). Cancer stem cells (CSCs) are a distinct population of cells inside tumors that possess the ability to undergo unlimited division and generate new tumors. These cells play a critical role in the progression, dissemination, and persistence of malignant tumors, as well as in the acquisition of resistance to cytotoxic treatment (28). The process of inducing a stem cell-like state in cancer cells is associated with an elevated level of resistance to therapy in tumor cells. The establishment of this state resembling stem cells is facilitated by cell-intrinsic proteins that are activated by senescence-associated secretory phenotypes (29). Subsequently, we proceeded to show the distribution of stemness scores and the distribution of bladder cancer samples. The former exhibited a range spanning from low to high values, while the latter depicted the samples in a sorted manner (**Figures 9G, H**). In summary, the findings of our study demonstrate a significant correlation between the presence of PPRF19 in bladder cancer specimens and the manifestation of a secretory phenotype often associated with senescence. There exists a potential regulatory relationship between PRPF19 and CCL16, which may contribute to the promotion of stemness in bladder cancer cells.

### 3.10 Construction of PRPF19-related ceRNA network

**Figure 10:**
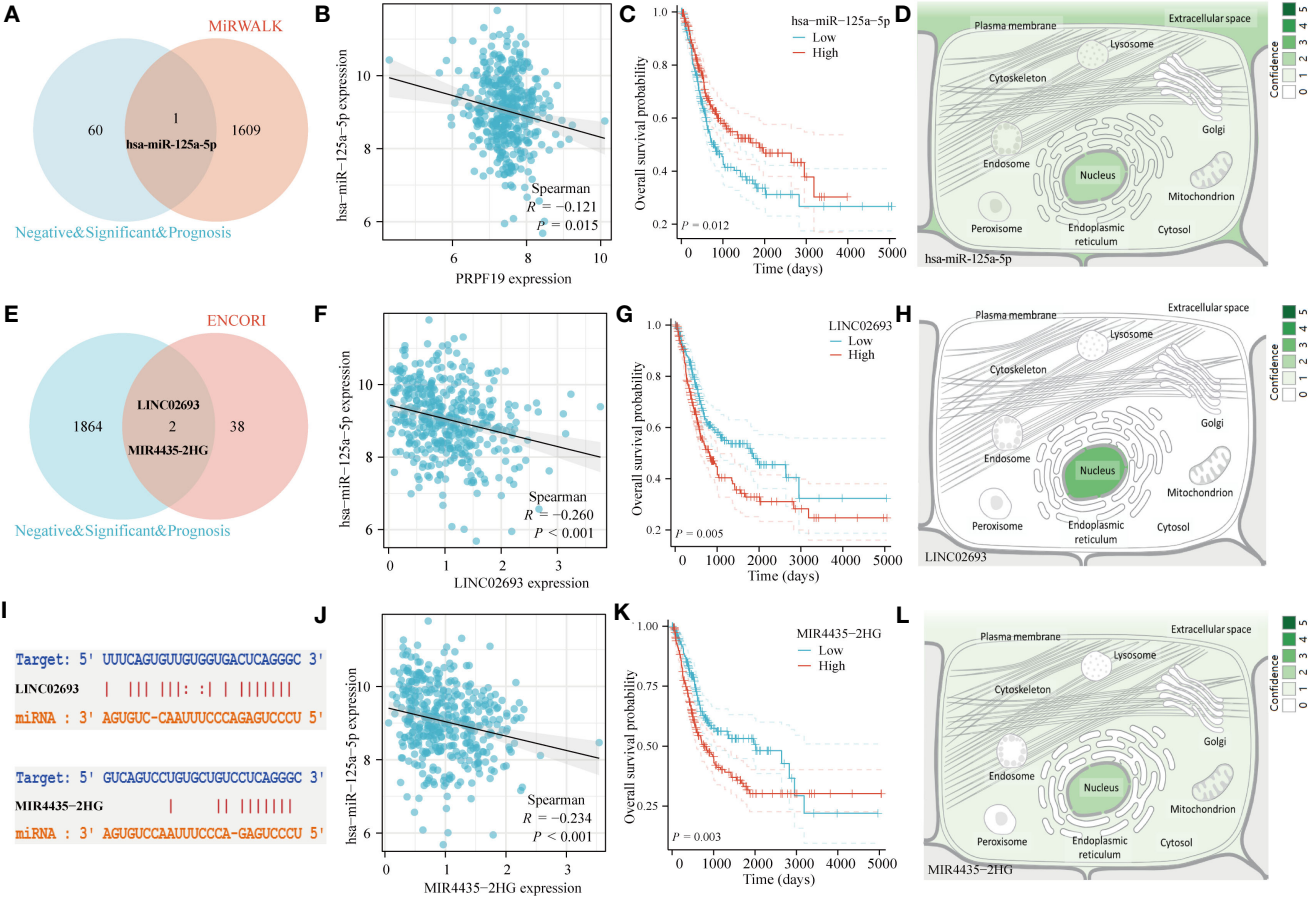
Construction of ceRNA network of PRPF19 in BLCA. **(A)** Venn diagram of miRNA interacting with PRPF19. **(B)** Scatter plot of PRPF19 and hsa-miR-125a-5p correlation. **(C)** Overall survival curves determined using Kaplan and Meier methods for hsa-miR-125a-5p. **(D)** The subcellular localization of miRNAs that were screened. **(E)** Venn diagram of LncRNA and hsa-miR-125a-5p interactions. **(F)** Scatter plot of LINC02693 and hsa-miR-125a-5p correlation. **(G)** Kaplan-Meier overall survival curves of LINC02693. **(H)** The subcellular localization of screened LncRNA. **(I)** Targeted binding sites of hsa-miR-125a-5p to LINC02693 and MIR4435-2HG. **(J)** Scatter plot of MIR4435-2HG and hsa-miR-125a-5p correlation. **(K)** MIR4435-2HG’s Kaplan-Meier overall survival curves. **(L)** The subcellular localization of LncRNA that were screened.

Multiple studies have shown the role of ceRNA in the process of tumor formation (30). This study conducted an analysis of the ceRNA network related with PRPF19 in BLCA. A total of 1610 target microRNAs for PRPF19 were first acquired from the MiRWALK website. The ceRNA hypothesis posits a negative link between the expression of microRNAs (miRNAs) and ceRNAs, such as messenger RNAs (mRNAs) and long non-coding RNAs (lncRNAs). Among the microRNAs examined, it was shown that only hsa-miR-125a-5p exhibited a substantial association with the prognosis of patients with bladder cancer. Additionally, this particular microRNA had a significant differential expression between bladder cancer tissues and normal tissues, as seen in **Figures 10A–C**. By using the Genecards online platform, we conducted a comprehensive analysis to determine the subcellular localization of hsa-miR-125a-5p. Our findings indicate that this microRNA mostly localizes inside the nucleus of cells, as seen in **Figure 10D**. Furthermore, the interaction between lncRNA and miRNA was shown using a Venn diagram. This was achieved by extracting miRNAs that were differently expressed from the ENCORI database. The elevated expression levels of LINC02693 and MIR4435-2HG were shown to be strongly linked to worse prognosis among individuals diagnosed with bladder cancer. Furthermore, their increased expression levels exhibited a negative correlation with hsa-miR-125a-5p. Consequently, these two LncRNAs were selected for further investigation. (**Figures 10E–G, I–K**). In addition, it was observed that both LINC02693 and MIR4435-2HG exhibited comparable localization inside the nucleus, as seen in **Figures 10H, L**. The improvement seen may be attributed to the PRPR19-related lncRNA, miRNA, and mRNA triple regulatory networks. These networks include the interaction of several RNAs, including LINC02693 and MIR4435-2HG, which act as ceRNAs to upregulate the expression of PRPF19 by sequestering hsa-miR-125a-5p.

## 4 Discussion

PRPF19, sometimes referred to as Pso4, SNEV, and NMP200, is situated on chromosome 11q12.2 (31). It is crucial in responding to DNA damage and processing mRNA. Various physiological processes that exert its influence include mRNA splicing and genome maintenance (32). In the context of cellular biology, it has been shown that PRPF19 has the ability to act as a stress response protein, hence enhancing the cellular capacity to withstand oxidative stress (33, 34). Furthermore, it has been found to have a role in mitigating apoptosis and DNA damage (35), as well as impeding cellular senescence (10). PRPF19, apart from its involvement in cellular biological mechanisms, exerts a significant regulatory function in many pathological conditions, such as cancers, by virtue of its multipotency. A research investigation revealed an upregulation of PRPF19 expression in hepatocellular carcinoma, which exhibited a strong association with worse disease prognosis (36). This work aimed to investigate the variations in PRPF19 expression in bladder cancer by using the BLCA dataset from the TCGA database, as well as the GSE13507 and GSE120736 datasets from the GEO database. Our analysis revealed that PRPF19 exhibited elevated expression levels in bladder cancer, with a notable increase seen in higher-grade tumors. This suggests that PRPF19 exerts an oncogenic function in the context of bladder cancer. In a similar vein, the findings of the prognostic analysis substantiated this assertion, as individuals exhibiting elevated levels of PRPF19 expression had a very unfavorable prognosis.

In order to investigate the probable mechanism behind the oncogenic involvement of PRPF19 in bladder cancer, a gene enrichment analysis was conducted. The MHC pathway was discovered by our research team as a potentially significant channel by which PRPF19 may exert regulatory control on the immune microenvironment in cases of bladder cancer. The complex biological interplay between immune cells inside the tumor microenvironment and tumor cells may give rise to diverse immunotherapeutic responses. Our investigation using single-cell RNA sequencing of BLCA tissues shown that PRPF19 exhibited predominant distribution in CD4 Tconv, CD8T, and NK cells. This finding suggests a potential association between PRPF19 and invading immune cells. The prognostic model used in our study utilized the LASSO algorithm and included eight essential immune infiltrating cells. Notably, individuals classified in the high-risk group had significantly worse prognoses compared to those in the low-risk category. The prognostic AUC values at 1, 3, and 5 years were all found to be higher than 0.6 in our model for patients with bladder cancer, as seen by the ROC curves.

There is an increasing body of data indicating that DNA methylation has an influence on the metabolic activities of cancer cells (37). DNA methylation plays a significant role in several metabolic processes, including glycolysis, the methionine cycle, and the tricarboxylic acid (TCA) cycle (38–40). In recent times, there has been a growing awareness of the potential use of DNA methylation status and its associated genes in the identification, management, and prediction of many illnesses (41, 42). In the present investigation, it was shown that there were significant variations in the methylation levels of PRPF19 between bladder cancer and normal bladder tissue. Hence, we conducted a more comprehensive examination of PRPF19 expression in bladder cancer by the utilization of methylation probes that possess predictive significance in this particular malignancy. In our investigation, it was shown that there exists a negative correlation between cg26375147 and PRPF19 in the context of bladder cancer. Additionally, a significant correlation was seen between cg26375147 and the prognosis of individuals diagnosed with bladder cancer. It is plausible that PRPF19 methylation may exert its influence on bladder cancer via the involvement of cg26375147.

The phenomenon of cellular senescence has been extensively acknowledged in the scientific community as a significant mechanism for the prevention of tumor growth (43). In previous studies, bladder cancer cells (44), colorectal cancer cells (45), and breast cancer cells (46) were subjected to cellular senescence, resulting in the induction of persistent cell cycle arrest and subsequent inhibition of growth in these tumor cells. While this strategy first shown efficacy in combating malignancies, it subsequently resulted in the development of tumor resistance as an unintended consequence. Senescent tumor cells do not die outright, but are transformed into tumor stem cells (47). The signaling pathways including p16, p21, and p53 are implicated in both senescence and stemness processes. This suggests that senescence could play a role in gene reprogramming and activation of stemness, hence contributing to tumor development driven by cancer stem cells (48). In this work, an investigation was conducted to examine the correlation between PRPF19 and genes linked to senescence-associated secretory characteristics across several bladder cancer datasets. The gene CCL16 was shown to have the strongest association with PRPF19 in the context of the senescence-associated secretory phenotype. According to recent research findings, it has been shown that the regulation of stemness in breast cancer cells is influenced by CCL16 (49). Furthermore, an examination was conducted to assess the significance of PRPF19 in relation to bladder tumor stem cells. The findings revealed that elevated expression of PRPF19 was associated with a greater stemness score. Hence, it is postulated that PRPF19 potentially modulates the stemness of bladder tumor cells by influencing the expression of the senescence-associated secretory phenotype-associated gene CCL16. The ceRNA network refers to a kind of post-transcriptional control that is facilitated by miRNAs. The task at hand is establishing the connection between the functions of coding and non-coding RNAs. The ceRNA network regulates the production of mRNA, potentially impacting several biological processes and contributing to the development of numerous diseases. This regulation occurs via the competitive binding of lncRNAs or circRNAs to miRNAs (50). In order to ascertain the presence of has-miR-125a-5p, an initial screening was conducted to find miRNAs that exhibited a connection or underwent survival analysis in conjunction with PRPF19. Two other LncRNAs were subjected to the same experimental methodology, leading to the identification of PRPF19, hsa-miR-125a-5p, LINC02693, and MIR4435-2HG as nuclear-localized entities. In summary, the construction of the ceRNA network aimed to elucidate the interaction profile of the BLCA gene. This was achieved by investigating the interactions involving PRPF19, hsa-miR-125a-5p, LINC02693, and MIR4435-2HG. This study analysed the role of PRPF19 in bladder cancer prognosis as well as in the immune microenvironment from several perspectives, but threw some limitations. We only confirmed the expression and prognostic value of PRPF19 in bladder cancer by bladder cancer tissue microarrays, and further experimental analysis of the role of PRPF19 and the immune microenvironment of bladder cancer is necessary, which will be further explored in our subsequent studies.

## 5 Conclusion

In summary, our study provides evidence that PRPF19 has a substantial impact on the immunological environment of bladder cancer and has the potential to serve as a prognostic indicator in patients afflicted with this condition. Additionally, it was shown that PRPF19 had a significant correlation with both the secretory phenotype often seen in senescence and the stemness characteristics of bladder cancer cells. Finally, it is worth noting that the ceRNA network under investigation has potential therapeutic implications in the context of bladder cancer.

## Data availability statement

The original contributions presented in the study are included in the article/supplementary materials. Further inquiries can be directed to the corresponding author.

## Ethics statement

The studies involving humans were approved by Shanghai outdo Ethical Committee. The studies were conducted in accordance with the local legislation and institutional requirements. The participants provided their written informed consent to participate in this study.

## Author contributions

YW: Conceptualization, Formal Analysis, Writing – original draft. JFW: Data curation, Writing – original draft. JH: Writing – original draft. BJ: Investigation, Writing – review & editing. ZP: Formal Analysis, Investigation, Writing – review & editing. JSW: Methodology, Writing – review & editing. YL: Supervision, Writing – review & editing. MR: Funding acquisition, Resources, Writing – review & editing.
